# Giant enhancement of third harmonic generation in an array of graphene ribbons using amplification of surface plasmon polaritons by optical gain

**DOI:** 10.1038/s41598-024-53493-3

**Published:** 2024-02-03

**Authors:** Marzieh Sedaghat Nejad, Abbas Ghasempour Ardakani

**Affiliations:** Department of Physics, School of Science, Shiraz University, Shiraz, 71946-84795 Iran

**Keywords:** Nonlinear optics, Optical properties and devices

## Abstract

In this paper, we theoretically study the enhancement of third-harmonic generation in a plasmonic structure composed of an array of trilayer graphene ribbons sandwiched between two $CaF_{2}$ layers. In fact, we suggest a new method for more enhancement of nonlinearity in plasmonic structures using incorporation of optical gain into graphene ribbons. As the pump intensity increases, the maximum output intensity of third harmonic generated (THG) wave versus fundamental frequency is blue-shifted while its value enhances. Our analysis indicates that the enhancement factor of THG in our proposed structure is 1.1 × 10^7^ without occurring an electric breakdown compared to case at which an optically pumped trilayer graphene sheet sandwiched between two CaF_2_ layers. Therefore, only presence of optical gain is not sufficient for significant enhancement of output intensity of THG wave and excitation of SPPs through the structure is also essential. On the other hand, our results demonstrate that the output intensity of THG wave from the proposed structure under optical pumping enhances by $10^{5}$ times compared to the plasmonic structure without optical gain which confirms the role of optical gain for THG enhancement in the plasmonic structure. This is because the gain in graphene ribbons amplifies the SPPs waves leading to the more field enhancement along the graphene ribbons which results in significant enhancement of THG wave in the plasmonic structure in comparison with one without gain. Therefore, we reveal that both SPPs and optical gain contribute to the strong output intensity of THG in our proposed structure compared to the trilayer graphene sheet inserted between two CaF_2_ layers.

## Introduction

Nonlinearity is an important concept with numerous applications in science and engineering, particularly in the fields of optics, photonics, signal processing^1,2^, and artificial intelligence^3,4^. Nonlinear optical devices such as frequency doublers, frequency quadruples, and harmonic generators are used in a variety of applications such as medical imaging^5,6^, remote sensing^7^, advanced spectroscopy, optical information processing and storage, light harvesting, bioimaging, integrated optics, and quantum technologies^8^. Nonlinearity of materials is typically observed at high light intensities because the photon-photon interaction is not strong enough to generate significant nonlinear effects. High light intensity is limited by material damage, thermal effects, beam quality, and cost. Therefore, enhancement of nonlinear effects such as harmonic generation and four wave mixing (FWM) using plasmonic structures has gained significant attention in recent years. For example, in 2016, Jin et. al. proposed a plasmonic metasurface based on silver nanostrips coupled to a metallic film to enhance FWM in a Kerr nonlinear material^9^. In another work, Mukhopadhyay et. al. experimentally demonstrated the dramatic enhancement of second harmonic generation (SHG) and third harmonic generation (THG) emissions in a gold grating by more than three orders of magnitude compared to a flat metal layer^10^. Although research on metallic plasmonic structures for enhancing nonlinearity is still ongoing, the efficiency of harmonic conversion cannot be significantly improved using metals due to their high intrinsic (ohmic) losses. Recently, graphene has gained significant attention as a potential material with large optical third order susceptibility $\chi^{\left( 3 \right)}$. The effective THz nonlinear optical susceptibility of the single-layer graphene was determined in 2018, which is significantly larger than the value found for other materials in the near infrared region by many orders of magnitude^11^. However, pumping waves with high powers are required to observe nonlinear effects of third order type in the graphene due to low thickness of graphene layers. Thus, a lot of attempts have been made to enhance nonlinear effects in graphene layers in recent years. In Ref.^12^, the integration of graphene with dielectric resonant waveguides leads to the enhancement of THG in graphene. In this work, the suggested structure resulted in significantly increased THG in the mid-infrared range due to the strong nonlinear interactions created by the confined field in the waveguide mode. The THG response of this structure was 9 orders of magnitude greater than that of graphene on a planar dielectric layer. In many papers, to produce the enhancement of THG, the electromagnetic field is confined through the graphene layer by excitation of graphene surface plasmons. In contrast to noble metals, graphene exhibits a robust plasmonic response in the far-infrared and THz frequency ranges with lower ohmic losses^13–15^. Furthermore, plasmonic properties of graphene can be tuned by applying an external electric or magnetic field^16,17^. Jan-Christoph has shown that graphene exhibited a significantly large nonlinear optical response in the terahertz range when it was coated with gold metallic gratings due to excitation of surface plasmon polaritons through the graphene layer^8^. In 2019, a nonlinear hybrid graphene-plasmonic structure was proposed to obtain an efficient THG response in THz frequencies. This nonlinear hybrid structure is based on a gold grating covered by a graphene layer. Strong coupling between the plasmonic resonance of the metallic grating and the surface plasmon along the graphene monolayer, resulted in a greatly enhanced electric field and increased light-matter interactions, leading to strong THG radiation^1^. Very recently, we proposed a random nonlinear plasmonic metasurface that consists of trilayer graphene deposited on a random grating. Our investigations indicated that the random structure exhibited a strong output intensity of THG wave and more field enhancement compared to the periodic structure due to Anderson localization of surface plasmon polaritons (SPPs) waves^19^. In some works, periodic nanostructures of graphene were used to excite graphene plasmons for enhancement of THG. In 2017, You et al. studied the nonlinearity of one- and two-dimensional graphene gratings made up of graphene ribbons and discs, respectively. They found that these plasmonic structures exhibited a strong enhancement of THG due to the double-resonant excitation of SPPs in graphene gratings^20^. Jin et al. proposed a Nonlinear Graphene-based metasurface composed of an array of graphene ribbons on top of a metal substrate and a Kerr nonlinear dielectric material sandwiched between them as a spacer layer. This structure exhibited high efficiency of THG response due to the excitation of SPPs along the surface of graphene and field enhancement at resonance mode^14^. In 2020, You et al. proposed a structure to enhance the THG in the graphene layer. In this scheme, the graphene nanoribbons with different widths were deposited on upper and lower facets of a dielectric slab. Because this structure supported plasmon resonance in both fundamental frequency and THG frequency, a dramatic enhancement of THG by 10^9^ was obtained compared to the graphene layer placed on the dielectric substrate^21^. In another work, Zhao et al. reported the enhancement of THG in sinusoidal curved graphene deposited on dielectric substrate up to a factor of 10^6^ compared to the graphene layer placed on a planar dielectric^22^. Furthermore, it was shown in Ref.^23^ that SPPs can be also excited at the interface between air and a multilayered structure composed of alternating semiconductor (InAS) and SiC dielectric layers in the presence of an external magnetic field in the terahertz region. It seems that the integration of graphene layers or ribbons with such metal-free metamaterials based on anisotropic semiconductor inclusions can be also used for increase of THG effect because of field enhancement due to the propagation of SPPs through the boundaries of this metamaterial.

Another way to increase the interaction of light with graphene is to utilize the optical gain in the graphene. In 2007, Ryzhii et al. suggested a graphene heterostructure to calculate the ac conductivity, which included both interband and intraband transitions, while considering the non-equilibrium electron–hole condition. Under strong optical pumping of the graphene layer, they showed that the net ac conductivity hence the absorption coefficient, could be negative in the terahertz range due to population inversion, leading to the amplification^24^. As a result, a single graphene layer can be used as a gain material to amplify electromagnetic waves. It is emphasized that the population inversion is necessary, but is not a guarantee of the optical gain in graphene due to Drude absorption by the carriers. The negative values of real part of the net dynamic conductivity, which includes both intraband and interband contributions, determine the gain at frequency ω^25^. In 2012, researchers proposed a THz amplifier to enhance gain with a resonant structure using the multi-layer graphene. Compared to amplifiers without the resonant structure, this amplifier demonstrated a significant improvement in emittance^26^. In 2022, we proposed a graphene-based terahertz plasmonic waveguide in which a graphene layer was deposited on a random grating in the presence of optical pumping. By using a random grating substrate, more amplification occurred in the resonance frequencies due to the multi-scattering. This resulted in an enhanced optical amplification of SPPs waves propagating along the graphene sheet^27^. Graphene exhibits a high plasmon gain value compared to the optical gain associated with stimulated emission of photons. This is due to its slow group velocity at terahertz frequencies and the strong confinement of the electromagnetic field in the vicinity of the graphene layer^28–31^. This property causes the structures based on optically pumped graphene layers to be ideal candidates for enhancing the nonlinearity.

As mentioned above, although various methods have been proposed recently to enhance nonlinear effects in graphene plasmonic structures, further efforts are still required to optimize and enhance the nonlinearity in the graphene layers. Therefore, in this study, we propose a plasmonic structure consisting of graphene ribbons on a $CaF_{2}$ substrate to achieve a highly effective third-harmonic generation process. For this purpose, we assume that the graphene ribbons are optically pumped to behave as a gain medium. When an electromagnetic wave is incident into the structure, it can generate a strong field enhancement due to the amplification of SPPs waves through the optically pumped graphene layer. This enhanced field can then improve the light-matter interaction, leading to a more efficient THG process. Thus, we indicate that the gain of graphene layer resulted from optical pumping provides another way to increase the output intensity of the THG wave in graphene based plasmonic structures.

### Designed structure and simulation method

**Figure 1:**
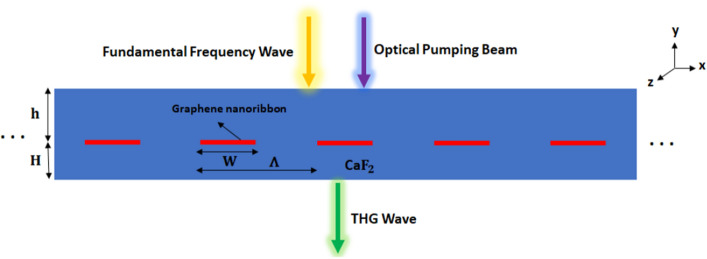
A schematic of the proposed nonlinear plasmonic structure based on the graphene ribbons sandwiched between two $CaF_{2}$ layers.

We propose a nonlinear plasmonic structure consisting of an array of periodic graphene ribbons sandwiched between two layers of $CaF_{2}$. Figure 1 shows the schematic of this structure. In this figure, the red horizontal line is a graphene ribbon and the blue layer presents $CaF_{2}$ with relative permittivity of ε = 1.7. The reason for using CaF_2_ in the structure instead of other substrates such as Si is its high electric breakdown field and lower relative permittivity. The thickness of $CaF_{2}$ layers located in the top and bottom of the graphene ribbons are h and H, respectively. The graphene ribbon array in the x-direction is located on the xz plane with the period Λ and width W. It is also assumed that the structure is embedded in the air. Thickness of the air layer above and below the structure is taken to be 350 nm and 500 nm, respectively in the simulation. We use finite element method (COMSOL Multiphysics) in 2D to compute linear and nonlinear responses of our proposed structure. The system is assumed to be infinite along the z-direction. Using periodic boundary conditions in the x-direction, only one unit cell is simulated. We employ the perfect matched layers (PMLs) on top and bottom of the structure to avoid unwanted reflections from the boundaries. Input and output ports are placed up and down in the y-direction. To calculate the output intensity of THG wave we use boundary prob in location of output port. The simulation parameters are chosen as: W = 1000 nm, Λ = 2000 nm, h = 700 nm and H = 500 nm.

According to our previous work^19^, the graphene is simulated as a surface current density. We use trilayer graphene instead of the graphene single layer because the excitation of SPPs through graphene few-layers is more convenient than one through the graphene single layer. For the Fermi energies lower than $\sqrt{2}{t}_{\perp }$, where ${t}_{\perp }\approx 0.4 \, {\text{eV}}$ is the trilayer hopping energy^32–34^, total surface current density of the ABA-stacked trilayer graphene is described as follows:

1 $$ \vec{J}(\omega ) = \vec{J}^{(1)} (\omega ) + \vec{J}^{(3)} (3\omega ) $$

The linear part of surface current density of graphene $\vec{J}^{(1)} (\omega )$ is given by: $\vec{J}^{(1)} \left( \omega \right) = 3\sigma^{\left( 1 \right)} \left( \omega \right)\vec{E}\left( \omega \right)$ where $\vec{E}\left( \omega \right)$ is the electric field of fundamental frequency along graphene surface. To describe the gain in the active graphene layer in the presence of optical pumping, we express the linear graphene conductivity $\sigma^{\left( 1 \right)} \left( \omega \right)$ as follows^27^:

2 $$ \begin{gathered} \sigma^{(1)} (\omega ) = (\frac{{e^{2} }}{4\hbar }) \times \left\{ {\frac{{8k_{B} T\tau }}{\pi \hbar (1 - i\omega \tau )}\ln \left[ {1 + \exp \left( {\frac{{E_{f} }}{{k_{B} T}}} \right)} \right] + \tanh \left( {\frac{{\hbar \omega - 2E_{f} }}{{4k_{B} T}}} \right) - \frac{4\hbar \omega }{{i\pi }}\int\limits_{0}^{\infty } {\frac{{G(\varepsilon ,E_{f} ) - G(\hbar \omega /2,E_{f} )}}{{(\hbar \omega )^{2} - 4\varepsilon^{2} }}} d\varepsilon } \right\} \hfill \\ \,\,\,\,\,\,\,\,\,\,\,\,\,\,\,\,\,\,\,\,\, \hfill \\ \end{gathered} $$

where e is the electron charge,$\hbar$ is the reduced Plank constant,$k_{B}$ is the Boltzmann constant, T = 300 K is the temperature,$\tau = 0.67\,{\text{ps}}$ is the electron and hole momentum relaxation time and $E_{f}$ is the quasi-Fermi energy. In Eq. (2), the function $G(\varepsilon ,E_{f} )$ is defined as follows^27^:

3 $$ G(\varepsilon ,\varepsilon^{\prime}) = \frac{{\sinh (\varepsilon /k_{B} T)}}{{\cosh (\varepsilon /k_{B} T) + \cosh (\varepsilon^{\prime}/k_{B} T)}} $$

It should be noted that in Eq. (2) the term $\tanh \left( {\frac{{\hbar \omega - 2E_{f} }}{{4k_{B} T}}} \right)$ results from the interband transition. It is well-known that the real part of interband conductivity, Re[σ_inter_], can be obtained from the following relation^35,36^:

4 $$ {\text{Re}} [\sigma_{{{\text{int}} er}} (\omega )] = (e^{2} /4\hbar )[f_{v} ( - \hbar \omega /2) - f_{c} (\hbar \omega /2)] $$

where $f_{v} (\varepsilon )$ and $f_{c} (\varepsilon )$ are the electron distribution in the valence and conduction bands respectively which are defined as follows:

5 $$ f_{\lambda } (\varepsilon ) = 1/\left( {1 + \exp \left( {\frac{{\varepsilon - \mu_{\lambda } }}{{k_{B} T}}} \right)} \right) $$

where $\mu_{\lambda } (\lambda = c,v)$ is the chemical potential corresponding to the conduction and valence bands. In equilibrium, we have $\mu_{c} = \mu_{v} = E_{f} \ge 0$ while for a non-equilibrium case such as population inversion with symmetrical pumping we have $\mu_{c} = - \mu_{v} = E_{f}$^35^. Therefore, for the case of population inversion resulting from optical excitation, substituting Eq. (5) in Eq. (4) leads to ${\text{Re}} [\sigma_{{{\text{inter}}}} ] = \tanh \left( {\frac{{\hbar \omega - 2E_{f} }}{{4k_{B} T}}} \right)$ appearing in Eq. (2). The details for derivation of other terms in Eq. (2) can be found in Ref.^36^. In the case of photoexcitation, $E_{f}$ depends on the intensity of optical pumping beam by the following relation^27,36^:

6 $$ E_{f} = \frac{{12\alpha \hbar^{2} v_{f}^{2} \tau_{R} }}{{\hbar \Omega k_{B} T}}I_{p} $$

where $\alpha = (e^{2} /\hbar ) \sim 1/137$ is the fine structure constant,$\tau_{R} = 10^{ - 7} \;{\text{s}}$ is the characteristic recombination time of the photogenerated electrons and holes, $v_{f} = 10^{6} \;{\text{m/s}}$ is the Fermi velocity. $I_{p}$ and $\hbar \Omega = 0.8\,{\text{eV}}$ are the intensity and photon energy of the incident optical pump radiation, respectively. In the ungated graphene layers and in the absence of pumping radiation, the Fermi energy is E_f_ = 0. It should be noted that Eq. (2) describes the conductivity of the graphene in which interband population inversion occurs by optical^36,37^ or electrical pumping^38,39^. In the case of electrical pumping, the Fermi energy is controlled by opposite gate voltages between split gates and metallic contacts in an electrically induced p–n junction^38^. However, in the case of optical excitation, the Fermi energy can be varied by frequency and intensity of the pumping radiation as observed in Eq. (6). When Fermi energy of graphene increases beyond a special value, the interband emission of photons becomes more than the intraband absorption leading to the negativity of real part of graphene conductivity. The frequency range at which Re(σ(ω)) becomes negative includes frequencies which fulfill the condition $\hbar \omega < 2E_{f}$. This frequency range widens with increase of Fermi energy or pumping intensity in the case optical pumping. Furthermore, the absolute value of Re(σ(ω)) increases with increase of Fermi energy or pumping intensity leading to the enhancement of gain coefficient of graphene layer in the terahertz frequency region. The reason for this behavior is that with increase of Fermi energy, the value of interband emission of photons enhances compared to the intraband absorption.

In Eq. (1), $\vec{J}^{(3)} (3\omega )$ is the surface current density of graphene at THG process. For ABA-stacked trilayer graphene $\vec{J}^{(3)} (3\omega )$ is expressed as follows provided Fermi energy is lower than $\sqrt{2}{t}_{\perp }$ as stated above:

7 $$ \vec{J}^{(3\omega )} \left( {3\omega } \right) = 3\sigma^{\left( 1 \right)} \left( {3\omega } \right)\vec{E}\left( {3\omega } \right) + \sigma^{\left( 3 \right)} \left( {3\omega } \right)\left( {\vec{E}\left( \omega \right) \cdot \vec{E}\left( \omega \right)} \right)\vec{E}\left( \omega \right) $$

where $\vec{E}\left( {3\omega } \right)$ is the electric field at the third harmonic frequency The third-order conductivity $\sigma^{\left( 3 \right)} \left( {3\omega } \right)$ is given by^40^:

8 $$ \sigma^{(3)} (3\omega ) = \frac{{3ie^{4} v_{f}^{2} }}{{4\pi \hbar^{2} E_{f} (\omega + i\tau^{ - 1} )(2\omega + i\tau^{ - 1} )(3\omega + i\tau^{ - 1} )}} $$

It should be noted that in Eqs. (1) and (7), the number of graphene layer is only multiplied to the linear conductivity while the nonlinear part is the same as one for graphene monlolayer. The reason for this behavior is that there are only one Dirac-type and two parabolic bands in the energy spectrum of the ABA-stacked graphene trilayer^32^. It is well-known that parabolic bands do not have any contribution in the THG response of graphene layers.

## Results and discussion

**Figure 2:**
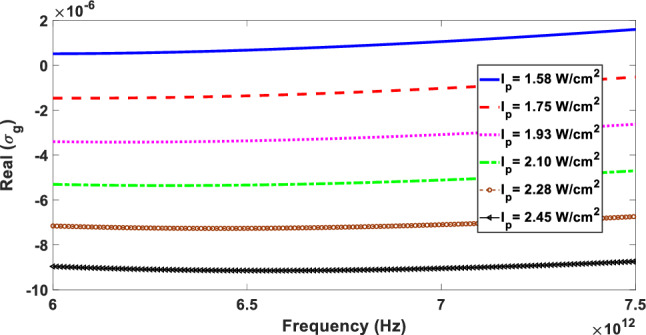
Real part of the linear conductivity of graphene as a function of frequency for different pump intensities with the same pump photon energy of $\hbar \Omega = 0.8\,{\text{eV}}$.

Because the real part of linear graphene conductivity is responsible for the optical gain or loss in graphene layers, we plot the real part of the graphene linear conductivity [real part of Eq. (2)] within the frequency range 6 to 7.5 THz in Fig. 2 at different pump intensities with the same pump photon energy of $\hbar \Omega = 0.8\,{\text{eV}}$. For each pump intensity, the Fermi energy is calculated according to Eq. (6). It can be observed that the real part of the graphene conductivity is dependent on the pump intensity and it becomes negative in a wide frequency range as the pump intensity increases from 1075 to $2.45\,\,{\text{W/cm}}^{2}$. The fact that the real part of the conductivity is negative under optical pumping enables graphene to act as a gain medium, amplifying propagating waves through it and enhancing light-matter interactions along the graphene layer at terahertz frequencies for the pump intensity in the range $1.75\,\,{\text{W/cm}}^{2}$ to $2.45\,\,{\text{W/cm}}^{2}$ at special frequency ranges. In addition, it is clearly seen from Fig. 2 that as the pump intensity increases, the value of the negative real part of graphene conductivity increases resulting in higher optical gain in the THz region. As a result, we expect that an optically pumped graphene layer shows stronger nonlinear effects in the THz region due to amplification of electromagnetic waves propagating through it provided the pump intensity exceeds a special value.

Now, it is assumed that the ribbon array in Fig. 1 is optically pumped with photon energy of $\hbar \Omega = 0.8\,{\text{eV}}$ and different pump intensities. We consider a TM polarized electromagnetic wave as the fundamental signal wave which is normally incident from upside onto the proposed structure in Fig. 1. The signal beam is taken to have the fixed input intensity of $12 \times 10^{3} \,\,{\text{W/cm}}^{2}$ and to be in the frequency range of 6 to 7.5 THz. The signal beam can excite SPPs waves through the graphene ribbon array due to the existence of periodicity.

**Figure 3:**
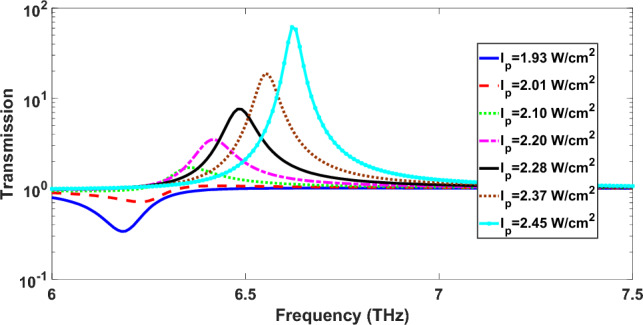
Transmission spectra of fundamental signal wave for the proposed nonlinear structure at different pump intensities in the range 1.93 to $2.45\,\,{\text{W/cm}}^{2}$. The parameters of the unit cell of structure are set as W = 1000 nm, Λ = 2000 nm, h = 700 nm and H = 500 nm.

We calculate the transmission spectrum of the signal wave at different pump intensities in the range 1.93 to $2.45\,\,{\text{W/cm}}^{2}$. The corresponding results are presented in Fig. 3 in a logarithmic scale for the transmittance axis. For pump intensities of 1.93 and $2.01\,\,{\text{W/cm}}^{2}$, there is a single dip in the transmission spectrum where its transmission increases with the pump intensity. This dip confirms the excitation of SPPs through the structure based on the array of graphene ribbons. In fact, the energy required for the excitation of SPPs is gained from the signal wave leading to the emergence of dip in the transmission spectrum. As the pump intensity increases, the value of transmittance in the dip increases due to the amplification of signal wave resulting from the amplification of SPPs waves. Another interesting feature in Fig. 2 is the blue-shift of resonance dip with increasing the pump intensity. This effect results from the increase of Fermi energy with increasing the pump intensity according to Eq. (6) and can be verified by the plotting the dispersion curve of SPPs propagating through the graphene layer as the Fermi energy increases.

When pump intensity further increases from 2.10 to $2.45\,\,{\text{W/cm}}^{2}$, in the transmission spectrum of signal wave, a peak appears such that the transmittance at peak increases with the increase of pump intensity. In addition, this single peak is blue-shifted as the pump intensity enhances. As shown in Fig. 3, for enough pump intensities, the value of transmittance is greater than 1 due to the amplification of fundamental signal wave and gain in the structure. Therefore, with increase of pump intensity from 2.10 to $2.45\,\,W/cm^{2}$, higher optical gain is available, resulting in the SPP waves to amplify more strongly. Consequently, the transmission spectrum exhibits a peak at the resonance frequency instead of a dip, as shown in Fig. 3.

**Figure 4:**
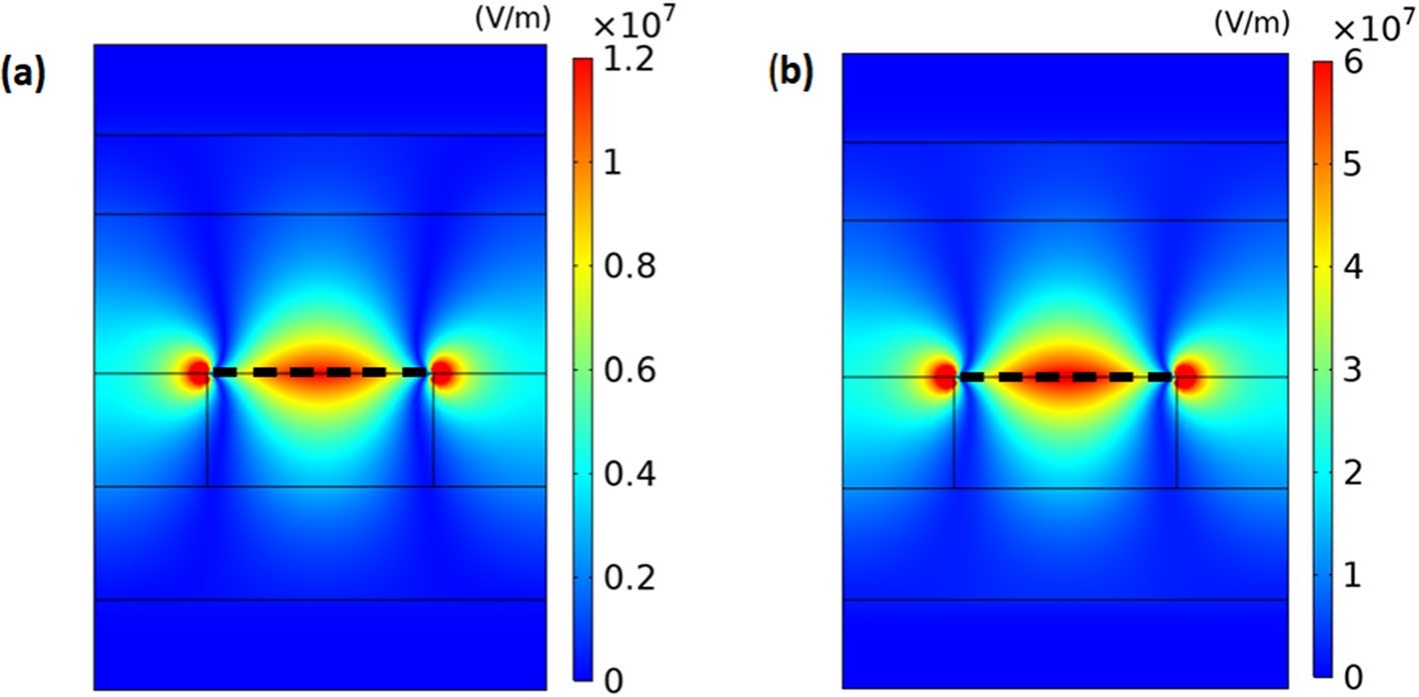
Distribution of absolute value of x-component of electric field in unit of V/m for fundamental signal wave at corresponding plasmon resonance frequency through the nonlinear plasmonic structure at pump intensities of (**a**) $I_{p} = 1.93\,\,{\text{W/cm}}^{2}$ and (**b**) $I_{p} = 2.45\,\,{\text{W/cm}}^{2}$. The thick dashed-line shows the graphene nanoribbon in the unit cell of the structure.

To confirm the excitation of SPPs through the graphene ribbon array, in Fig. 4, we display the distribution of absolute value of x component of the electric field corresponding to the fundamental signal wave for pump intensities of $I_{p} = 1.93\,\,{\text{W/cm}}^{2}$ and $I_{p} = 2.45\,\,{\text{W/cm}}^{{2}}$ which correspond to the minimum dip and maximum peak in the transmission spectrum of signal wave in Fig. 3, respectively.

It is evident from the Fig. 4 that the electric field is mainly localized around the interface along the graphene ribbon where the SPPs wave is excited. This figure shows that the maximum electric field value for $I_{p} = 2.45\,\,{\text{W/cm}}^{2}$ is approximately 5 times greater than that for $I_{p} = 1.93\,\,{\text{W/cm}}^{2}$. This is primarily due to the fact that as the pump intensity increases, the real conductivity of graphene also increases, as illustrated in Fig. 2. Consequently, the SPPs propagating through the graphene ribbon are also amplified, resulting in stronger electromagnetic fields. This amplification can improve the light-matter interaction, leading to an enhanced nonlinearity response.

In order to show theoretically the excitation of SPPs through the structure, we use the dispersion relation of SPPs propagating through the graphene layer. When a graphene layer or a multilayer is located at the interface between two infinite media with relative electric permittivity of $\varepsilon_{r1}$ and $\varepsilon_{r2}$, the dispersion relation for TM polarized graphene plasmons is given by^41^:

9 $$ \frac{{\varepsilon_{r1} }}{{\sqrt {\beta^{2} - \varepsilon_{r1} \frac{{\omega^{2} }}{{c^{2} }}} }} + \frac{{\varepsilon_{r1} }}{{\sqrt {\beta^{2} - \varepsilon_{r1} \frac{{\omega^{2} }}{{c^{2} }}} }} = - \frac{i\sigma (\omega )}{{\omega \varepsilon_{0} }} $$

where $\beta$ is the propagation constant of graphene plasmons and $\sigma (\omega )$ is the graphene conductivity. Because the periodicity ($\Lambda$) and strip width of graphene microribbons (w) are much smaller than the wavelength of the incident FF wave, the array of trilayer graphene ribbons in Fig. 1 can be treated as a surface with an effective conductivity as follows^42^:

10 $$ \sigma_{eff} (\omega ) = 3\frac{w}{\Lambda }\sigma^{(1)} (\omega ) $$

**Figure 5:**
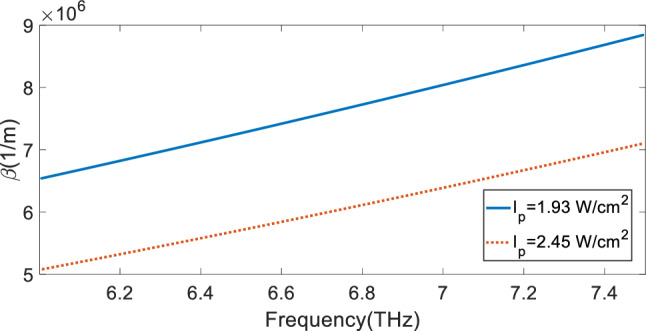
Dispersion curves for SPPs propagating through an array of trilayer graphene sandwiched between two semi-infinite CaF_2_ media with the relative permittivity ε = 1.7 at different pumping intensities I_p_ = 1.93 W/cm^2^ (blue solid-line curve) and I_p_ = 2.45 W/cm^2^ (red dotted-line curve).

By substituting Eqs. (10) into (9) and taking $\varepsilon_{r1} = \varepsilon_{r2} = \varepsilon_{{CaF_{2} }} = 1.7$, we obtain the dispersion curve of graphene plasmons excited by FF incident wave. For two pumping intensity of 1.93 W/cm^2^ and 2.45 W/cm^2^, the dispersion curves are calculated and the corresponding results are shown in Fig. 5. For the case I_p_ = 1.93 W/cm^2^, from the blue solid-line dispersion curve in Fig. 5 for the frequency 6.2 THz corresponding to the dip of blue solid-line curve in Fig. 3, the value of propagation constant of graphene plasmon is obtained as $\beta = 6.80 \times 10^{6} \,1/{\text{m}}$. When a grating is used for excitation of SPPs, the phase matching condition for a beam at normal incidence onto the grating with period Λ is $k_{sp} = \frac{2\pi }{\Lambda }q$, where q is an integer denoting the diffraction order and k_sp_ is the propagation constant of SPPs. For the proposed structure in Fig. 1, the array of graphene ribbons with period of Λ = 2000 nm also acts as a grating for the excitation of graphene plasmons. For q = 2, the propagation constant provided by the grating is $k_{sp} = 2\pi \times 10^{6} \,1/{\text{m}} \simeq 6.28 \times 10^{6} \,1/{\text{m}}$ which is close to the value $\beta = 6.80 \times 10^{6} \,1/{\text{m}}$ obtained from the dispersion curve. In the same way, for the case I_p_ = 2.45 W/cm^2^, at frequency f = 6.62 THz corresponding to the maximum peak in Fig. 3, from the red dotted-line dispersion curve in Fig. 5, the propagation constant of graphene plasmon is $\beta = 5.90 \times 10^{6} \,1/{\text{m}}$ which is close to $k_{sp} = 2\pi \times 10^{6} \,1/{\text{m}} \simeq 6.28 \times 10^{6} \,1/{\text{m}}$. The difference between the propagation constant obtained from the dispersion curve and one obtained from the phase matching condition can be due to the finite thickness of CaF_2_ layer in the simulated structure and the error due to using the effective conductivity approximation in Eq. (9). Therefore, the frequency of dips and peaks in Fig. 3 are matched to the dispersion curve of graphene surface plasmons which provides another reason for excitation of graphene SPPs and their role in the increase of transmission above 1 in Fig. 3.

**Figure 6:**
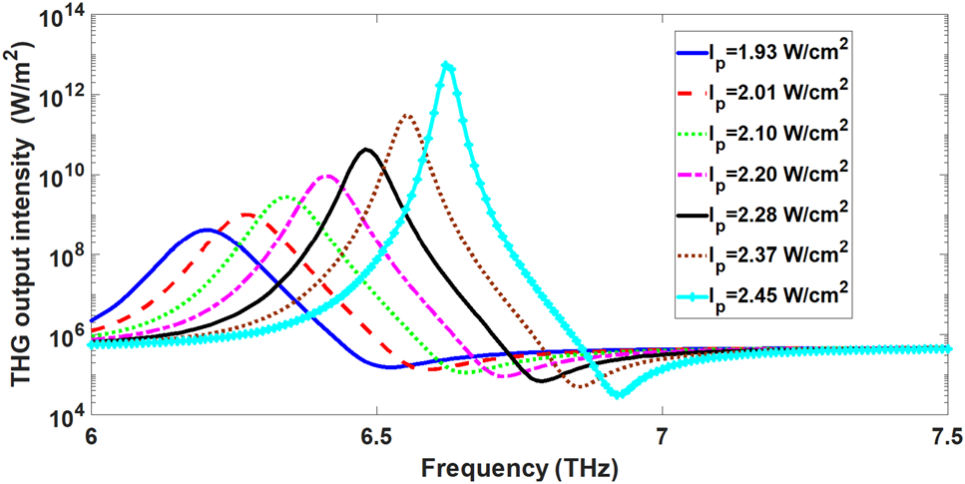
THG output intensity from the proposed nonlinear plasmonic structure versus the fundamental frequency of input signal wave at different pump intensities in the range 1.93 to $2.45\,\,{\text{W/cm}}^{2}$.

In order to study the nonlinear effect in the proposed plasmonic structure, we investigate the THG process in this plasmonic structure. We calculate the output intensity of THG wave for input intensity of fundamental signal wave $12 \times 10^{3} \,\,{\text{W/cm}}^{2}$ under different pump intensities as a function of frequency of fundamental signal wave. To obtain the output intensity of THG wave, we calculate the component of time-averaged Poynting vector in the − y direction $\left( {\vec{S}_{av} \cdot \left( { - \hat{y}} \right) = \frac{1}{2}{\text{Re}} [\vec{E} \times \vec{H}^{ * } ] \cdot \left( { - \hat{y}} \right)} \right)$ at the output port as in Ref.^18^. The corresponding results are displayed in Fig. 6 whose vertical axis is shown in a logarithmic scale. The other parameters are the same as those in Fig. 3. The output intensity of THG signal becomes maximum in the vicinity of resonance frequency in the transmission spectrum of input signal wave. By increasing the pump intensity, we find that the output intensity of the THG wave shifts to higher frequencies. This means that, similar to the transmission spectrum, the frequencies that exhibit the maximum third harmonic generation can be manipulated by adjusting the pump intensity. This effect is due to the blue shift of plasmon resonance frequency with increase of pump intensity as observed in Fig. 3. As illustrated in Fig. 6, when the pump intensity increases from 1.93 to $2.45\,\,{\text{W/cm}}^{2}$, the maximum output intensity of the THG wave dramatically increases from $4.19 \times 10^{8} \,\,{\text{W/m}}^{2}$ to $5.46 \times 10^{12} \,\,{\text{W/m}}^{2}$. Therefore, in Fig. 6, the strongest output intensity for THG wave is observed at the fundamental frequency of 6.62 THz at pump intensity of $2.45\,\,{\text{W/cm}}^{2}$ which is approximately $1.3 \times 10^{4}$ time greater than the maximum of output intensity of THG wave at pump intensity of $1.93\,\,{\text{W/cm}}^{2}$ at fundamental frequency of 6.21 THz. Therefore, enhancement of intensity of the beam employed for optical pumping the graphene layers or ribbons in plasmonic structures provides another tool to further enhance THG effect.

**Figure 7:**
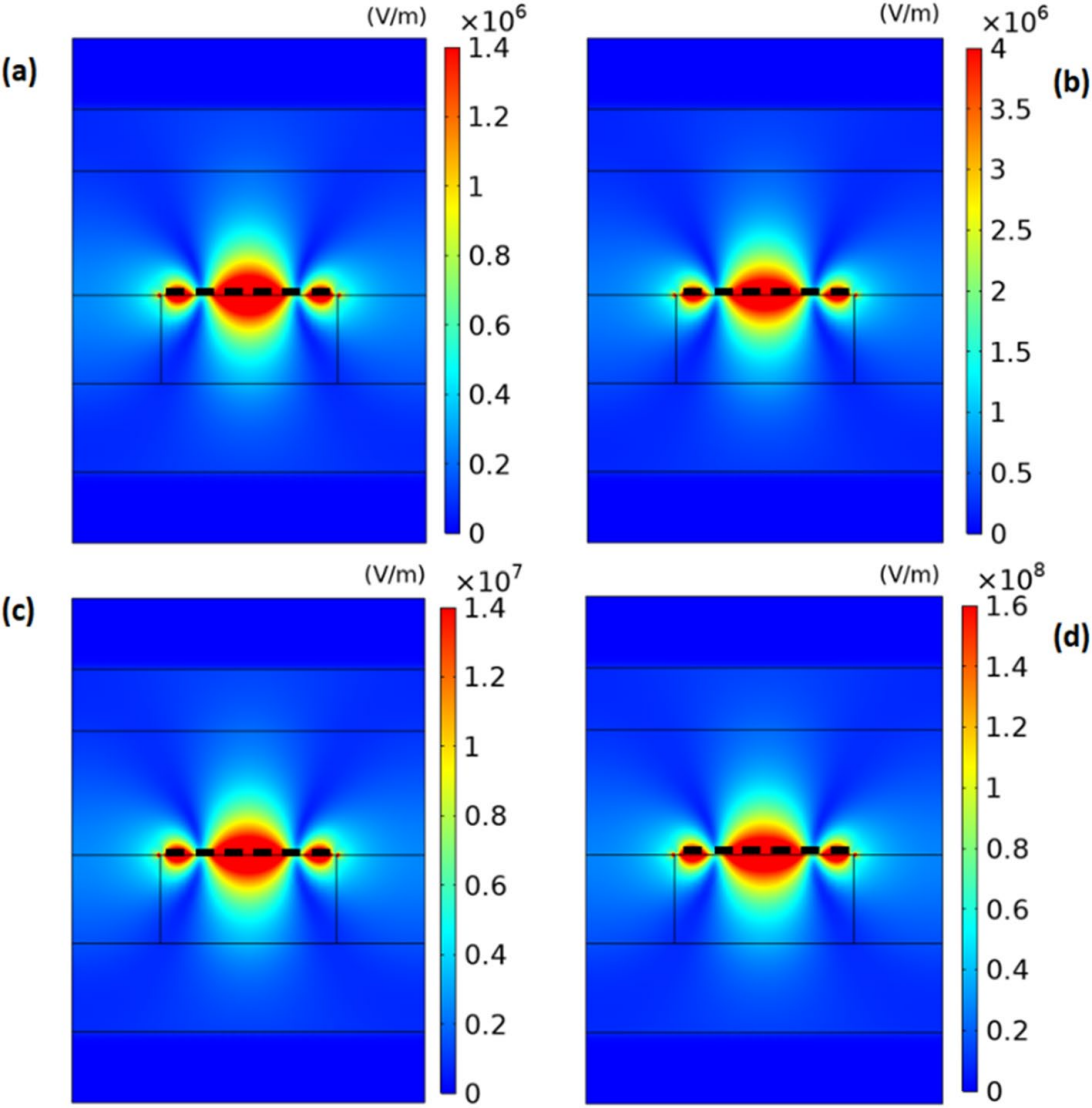
The distribution of absolute value of x component of electric field of maximum value of THG wave in unit of V/m through the nonlinear structure obtained under illumination of fundamental signal wave and under different pump intensities of (**a**) $I_{p} = 1.93\,\,{\text{W/cm}}^{2}$, (**b**) $I_{p} = 2.10\,\,{\text{W/cm}}^{2}$, (**c**) $I_{p} = 2.28\,\,{\text{W/cm}}^{2}$ and (**d**) $I_{p} = 2.45\,\,{\text{W/cm}}^{2}$. The thick dashed line shows the graphene nanoribbon.

To better understand the mechanism for enhancement of output intensity of THG wave with increasing the pump intensity, we display the distributions of the absolute value of x component of electric field for THG wave at pump intensities of $I_{p} =$ 1.93, 2.10, 2.28 and $2.45\,\,{\text{W/cm}}^{2}$, and show the corresponding results in Fig. 7. As shown in this figure, the electric field for THG wave is localized around the graphene ribbon and it is diminished in the left- and right-hand side of the graphene ribbon because nonlinear effect exists only in graphene nanoribbons. In Fig. 7, there are a high intensity region with a larger length and two ones with smaller lengths along the graphene ribbon while in Fig. 4 there is only one high intensity region in the graphene ribbon. The reason for this behavior is that the frequency in Fig. 7 is equal to three times of one in Fig. 4. Another observed interesting feature in Fig. 7 is that the maximum value of electric field of THG wave increases from $1.4 \times 10^{6} \,\,{\text{V/m}}$ to $1.6 \times 10^{8} \,\,{\text{V/m}}$ with increase of pump intensity from 1.93 to $2.45\,\,{\text{W/cm}}^{2}$. This effect leads to the enhancement of output intensity of THG wave which exits from the downside of the structure with increasing the pump intensity. By increasing the pump intensity, SPPs excited by input fundamental signal wave at the graphene ribbon array are more amplified due to higher gain in graphene. When the maximum value of x component of electric field of fundamental signal wave increases by 5 times with increase of pump intensity from 1.93 to $2.63\,\,{\text{W/cm}}^{{2}}$ in Fig. 4, According to Eq. (7), the maximum value of surface density $\overrightarrow {J}^{(3)} (3\omega )$ increases by 125 times. Therefore, we expect that the electric field corresponding to THG wave increases by 125 times. But in Fig. 7 we see that the electric field increases by 114 times. The reason for less enhancement than 125 times in Fig. 7 is that due to blue-shift of plasmon resonance frequency with increase of pump intensity, the field of THG in Fig. 7d with frequency of 3f = 19.86 THz experiences more optical loss than the field in Fig. 7a with frequency of 3f = 18.63 THz. In addition, according to Eq. (8), the third-order conductivity of graphene is inversely proportional to E_f_ or intensity of optical pumping wave. This effect also leads to the less enhancement than 125 times in Fig. 7. Because the output intensity of THG wave is proportional to square of electric field intensity, we expect that output intensity of THG increases by $114^{2} = 12,996$ which is close to the enhancement of output intensity of THG by $13 \times 10^{3}$ in Fig. 6 as the pump intensity increases from 1.93 to $2.45\,\,{\text{W/cm}}^{2}$.

Thus, THG effect becomes more pronounced as the electric field of SPPs wave increases. As a result, we expect that the electromagnetic fields of the THG wave increase with increase of pump intensity. It should be noted that the direct amplification due optical gain does not occur for THG fields because the frequency of THG wave (3f where f is the frequency of fundamental signal wave) is not within in the frequency range at which the real part of graphene linear conductivity is negative.

As the pump intensity increases at a fixed input intensity of signal wave, the output intensity of THG wave also increases and therefore the electric field corresponding to both fundamental signal and THG waves increases around the graphene ribbon in the structure. However, it is important to note that the pump intensity does not increase without bound because the electric field in the structure should be lower than the breakdown field of the materials used in the structure. The results presented in Figs. 4 and 7 show that the maximum absolute value of the electric field is $$6 \times 10^{7}$$ and $$1.6 \times 10^{8} \,{\text{V/m}}$$, respectively, which is below the dielectric strength of $$CaF_{2}$$$$(3 \times 10^{8} \,{\text{V/m}})$$^43,44^. As a result, there is no electric breakdown in this structure.

**Figure 8:**
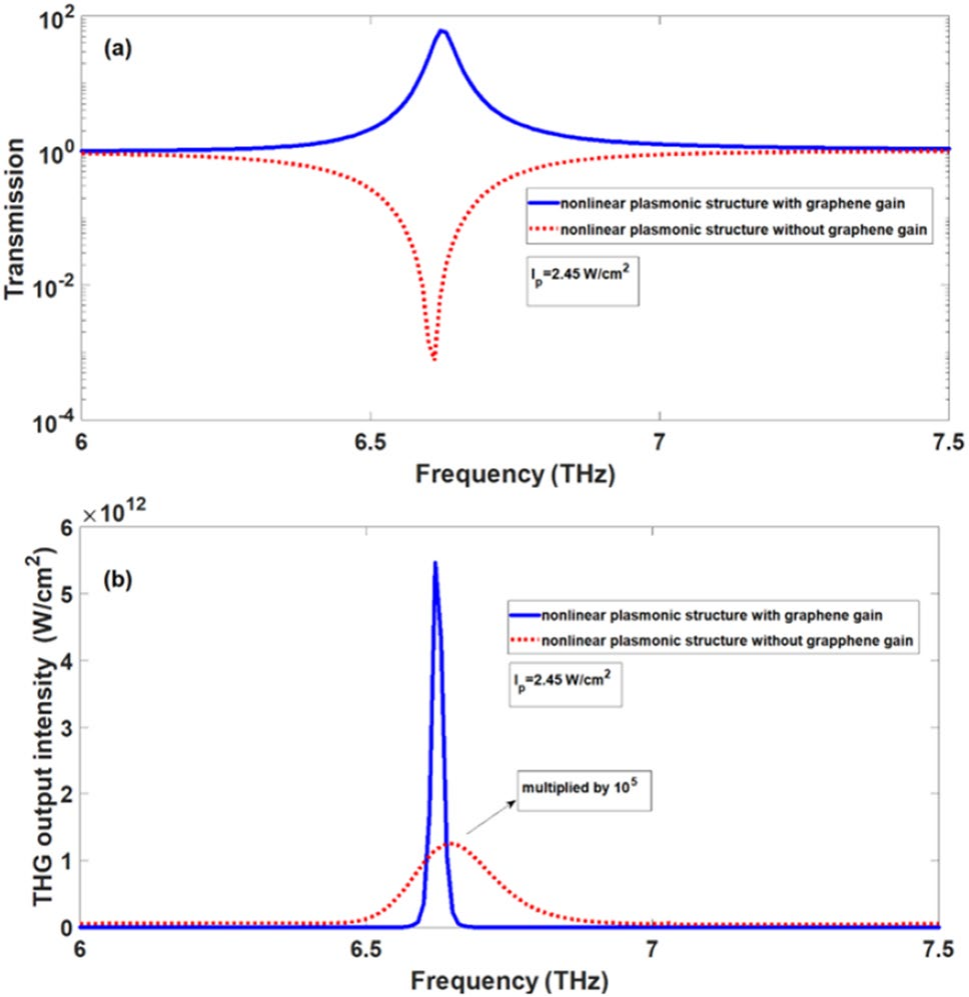
(**a**) transmission spectra and (**b**) THG output intensity of the nonlinear plasmonic structure, when the gain in graphene ribbons is included (blue solid-line) and the gain in graphene ribbons is ignored (red dotted-line) at $I_{p} = 2.45\,\,{\text{W/cm}}^{2}$.

It should be noted that the significant enhancement of THG in Fig. 6 is due to the presence of graphene SPPs as well as optical gain of graphene ribbons. To clearly indicate the effect of optical gain in the graphene ribbons on the enhancement of THG, in Fig. 8a, we compare the transmission spectrum for the case at which the ribbon array has optical gain at pump intensity of $2.45\,\,{\text{W/cm}}^{2}$ (blue solid-line) with the case at which real part of linear graphene conductivity of the graphene ribbons is neglected (red dotted-line) so they have no optical gain or loss. It is clearly seen in Fig. 8a that for the case at which graphene ribbons have optical gain, one peak exists in the transmission spectrum whose value is larger than 1 confirming the amplification in the structure. In this case, the SPPs waves can be amplified. However, for the case at which the graphene ribbons have no optical gain or loss, there is one dip in the transmission spectrum. In this case, no amplification occurs for SPPs waves and they only can be excited through the structure. In Fig. 8b, output intensities of THG wave as a function of frequency of incident signal wave for the case in which the ribbons have optical gain (blue solid-line) and the case of ribbons without gain (red dotted-line) are presented.

The output intensity of the THG wave in the nonlinear structure including the optical gain in the graphene ribbons is about $4 \times 10^{5}$ times greater than the case without the optical gain of graphene ribbons. Therefore, substantial role of optical gain in the enhancement of THG effect is confirmed from the results shown in Fig. 8b.

**Figure 9:**
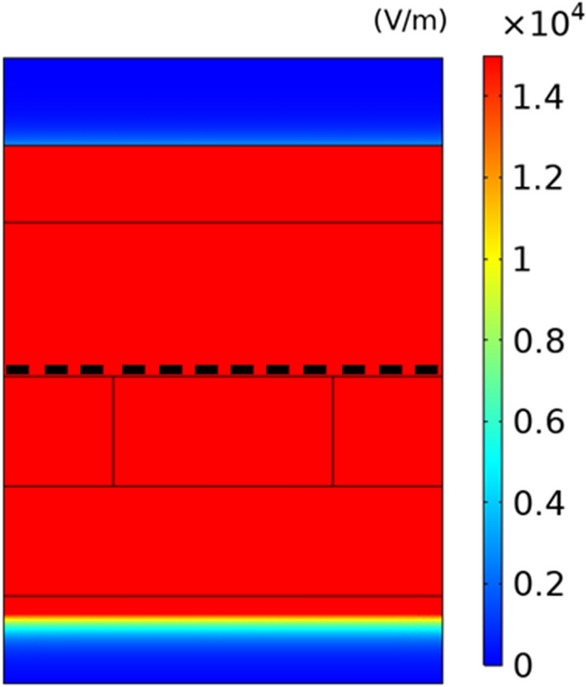
The distribution of absolute value of x component of electric field of fundamental signal wave in unit of V/m through the nonlinear structure containing graphene layer instead of the graphene ribbon array obtained under illumination of fundamental signal wave and under the pump intensity of $I_{p} = 2.45\,\,{\text{W/cm}}^{2}$ at fundamental frequency of 6.62 THz. The thick dashed-line shows the graphene layer.

To demonstrate the role of excitation of SPPs in enhancing the output intensity of THG wave, it is assumed that in Fig. 1 a graphene layer with width of 2000 nm covers the whole of $CaF_{2}$ substrate in the unit cell. In this case because the phase matching condition is not satisfied, no SPPs waves are excited through the graphene layer. The distribution of x component of electric field intensity corresponding to fundamental signal wave is shown in Fig. 9. In this figure, it is assumed that the graphene layer is pumped with pump intensity of $2.45\,\,\,{\text{W/cm}}^{2}$. One can see in Fig. 9 that no field localization occurs around the graphene layer, so there is no evidence for excitation of SPPs through the graphene layer.

**Figure 10:**
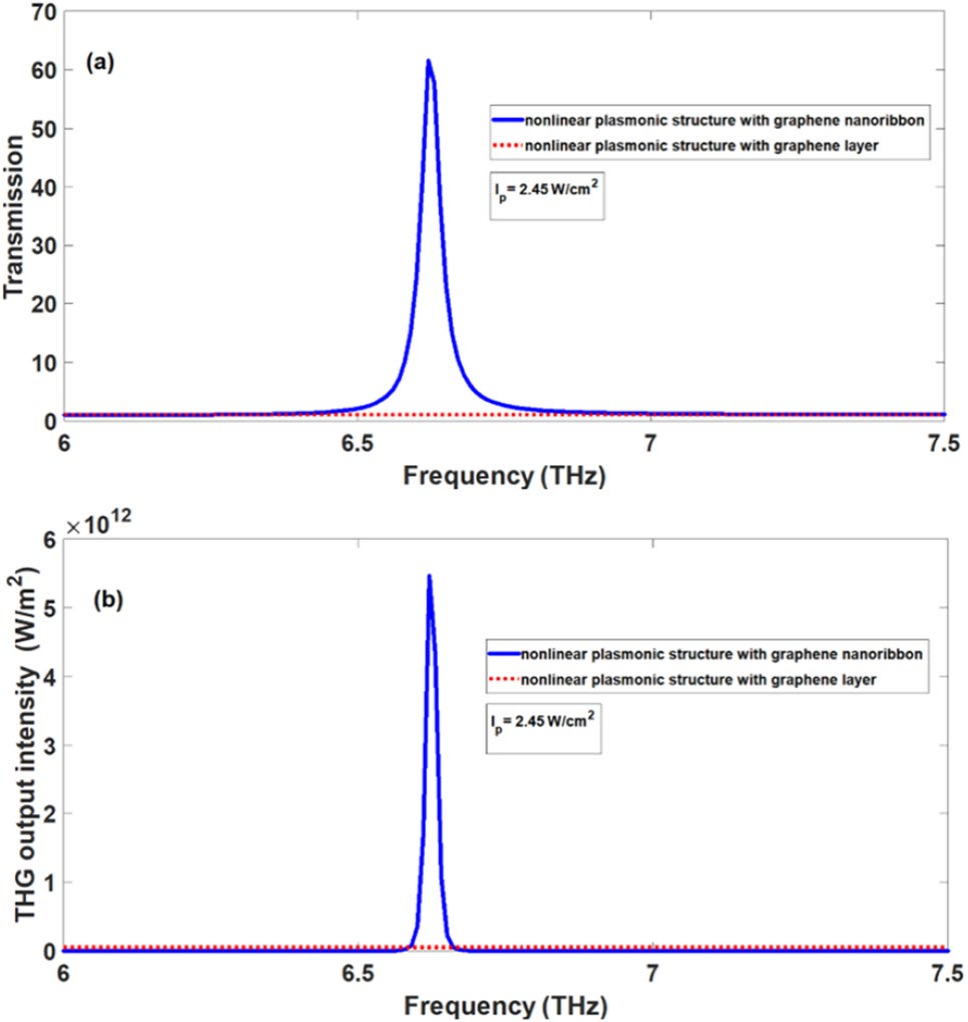
(**a**) transmission spectra and (**b**) THG output intensity of the nonlinear plasmonic structure, for the structure based on graphene layer (red dotted-line) and for the structure based on the graphene ribbon array (blue solid-line) at the same pump intensity of $I_{p} = 2.45\,\,{\text{W/cm}}^{2}$.

In addition, in Fig. 10a, we plot the transmittance of the signal wave through the structure corresponding to the structure in Fig. 9 (red dotted-line curve). It is clearly seen in this case that the transmittance is approximately equal to one and there is no dip or peak in the transmission spectrum confirming the absence of SPPs excitation. For more comparison, the transmittance curve for the structure shown in Fig. 1 is displayed in Fig. 10a at pump intensity of $2.45\,\,{\text{W/cm}}^{2}$ (blue solid-line curve). Hence, when SPPs are excited in the structure with graphene ribbons in the presence of optical gain, significant amplification occurs for SPPs wave leading the emergence of one peak in the transmission spectrum with transmittance T ~ 61.5. Furthermore, in Fig. 10b, we plot the output intensity of THG wave for the structure corresponding to Fig. 9 (red dotted-line curve) and compare with the output intensity of THG for the structure containing graphene ribbons (blue solid-line curve). In both curves of Fig. 10b, the graphene layer and graphene ribbons are optically pumped with the same pump intensity of $2.45\,\,{\text{W/cm}}^{2}$. For the case of structure with ribbons, the output intensity of THG by 1.1 × 10^7^ is higher than one in the case of structure based on graphene layer, although two structures are optically pumped with the same intensity. This effect is attributed to the fact that in the case of structure containing graphene ribbons, presence of SPPs leads to the field enhancement effect and stronger output intensity for THG wave. From comparison of two curves in Fig. 10b one can deduce that the presence of SPPs is essential for significant enhancement of THG effect and only the presence of optical gain is not sufficient to obtain strong output intensity of THG. However, existence of optical gain in graphene based plasmonic structures leads to further enhancement of THG process compared to one without optical gain.

It should be noted in works such as Ref.^21^ there were no reports for the maximum value of electric field in the structures and the authors did not consider whether the electric breakdown occurred in the structure or not. In our proposed structure in this paper, at a higher pump intensity of $2.55\,\,{\text{W/cm}}^{2}$, it is possible to enhance THG by 10^9^ compared to the graphene sheet sandwiched between two CaF_2_ layers (This enhancement factor is the same as one obtained in Ref.^21^). However, in this case the maximum value of electric field is greater than the electric breakdown of CaF_2_.

Furthermore, introduction of optical gain in previous plasmonic structures proposed for enhancement of THG through the graphene layer such as Ref.^19,21,22^ can lead to more enhancement of THG. But we should take care that the electric field does not surpasses the breakdown field.

It is worth mentioning that the enhancement of THG in a graphene layer using an array of gold nano-ribbons has been experimentally observed in Ref.^45^. In this work, it was shown that the THG intensity increased by three orders of magnitude compared to the bare graphene due to the excitation of graphene surface plasmons in the presence of gold grating deposited on top of the graphene layer in the frequency range 55 to 77 THz. Furthermore, in recent years, some experimental works have been performed for achieving terahertz lasers and amplification of terahertz waves using graphene layers^46,47^. Consequently, the experimental realization of the proposed structure in this paper can be achieved in the future.

## Conclusion

In this paper, we have numerically studied the effect of optical gain on the THG in a nonlinear plasmonic structure composed of the array of trilayer graphene ribbons sandwiched between two $CaF_{2}$ layers. In this system, both optical gain and nonlinearity are provided by trilayer graphene ribbons. The maximum output intensity for THG wave occurs in the surface plasmon resonance frequency corresponding to the fundamental signal wave. The peak of output intensity curve versus the frequency of fundamental signal wave is blue-shifted with increase of pump intensity while its value enhances. By increasing the pump intensity from 1.93 to $2.45\,\,{\text{W/cm}}^{2}$, the output intensity of THG increases from $4.19 \times 10^{8}$ to $5.46 \times 10^{12} \,\,{\text{W/m}}^{2}$, while the resonance frequency moves from 6.21 to 6.62 THz. Our results confirm that THG in the proposed structure is 10^5^ times larger compared to the case at which the gain of graphene ribbons is ignored. In addition, THG in our proposed structure is 1.1 × 10^7^ larger than the structure at which an optical pumped trilayer graphene sheet is sandwiched between two CaF_2_ layers confirming the simultaneous presence of both SPPs waves and optical gain is required to significantly enhance THG process. In other words, graphene based plasmonic structures which do not support the propagation of SPPs are not efficient platforms for strong THG even if they are optically pumped to have gain. Consequently, our results reveal that optical pumping the graphene ribbons in nonlinear plasmonic structures which guarantee the propagation of SPPs is a promising tool for further enhancement of output intensity of THG wave compared to the similar plasmonic structure without gain. It should be noted that more enhancement of THG obtained from the proposed structure is possible but the electric field inside the structure becomes greater than the electric breakdown. This effect was not taken into account in some previous works where the enhancement factor of THG has been reported greater than 10^7^.

## Data Availability

The data that support the findings of this study are available from the corresponding author upon reasonable request.
